# Degraded and computer-generated speech processing in a bonobo

**DOI:** 10.1007/s10071-022-01621-9

**Published:** 2022-05-20

**Authors:** Nicole J. Lahiff, Katie E. Slocombe, Jared Taglialatela, Volker Dellwo, Simon W. Townsend

**Affiliations:** 1 Department of Psychology, University of York, York, UK; 2 Department of Ecology, Evolution and Organismal Biology, Kennesaw State University, Kennesaw, USA; 3 Ape Cognition and Conservation Initiative, Des Moines, USA; 4 Department of Computational Linguistics, University of Zurich, Zurich, CH Switzerland; 5 Center for the Interdisciplinary Study of Language Evolution, University of Zurich, Zurich, Switzerland; 6 Department of Comparative Language Science, University of Zurich, Zurich, CH Switzerland; 7 Department of Psychology, University of Warwick, Coventry, UK

**Keywords:** Degraded speech, Speech evolution, Bonobo, Kanzi, Comparative approach

## Abstract

The human auditory system is capable of processing human speech even in situations when it has been heavily degraded, such as during noise-vocoding, when frequency domain-based cues to phonetic content are strongly reduced. This has contributed to arguments that speech processing is highly specialized and likely a de novo evolved trait in humans. Previous comparative research has demonstrated that a language competent chimpanzee was also capable of recognizing degraded speech, and therefore that the mechanisms underlying speech processing may not be uniquely human. However, to form a robust reconstruction of the evolutionary origins of speech processing, additional data from other closely related ape species is needed. Specifically, such data can help disentangle whether these capabilities evolved independently in humans and chimpanzees, or if they were inherited from our last common ancestor. Here we provide evidence of processing of highly varied (degraded and computer-generated) speech in a language competent bonobo, Kanzi. We took advantage of Kanzi’s existing proficiency with touchscreens and his ability to report his understanding of human speech through interacting with arbitrary symbols called lexigrams. Specifically, we asked Kanzi to recognise both human (natural) and computer-generated forms of 40 highly familiar words that had been degraded (noise-vocoded and sinusoidal forms) using a match-to-sample paradigm. Results suggest that—apart from noise-vocoded computer-generated speech—Kanzi recognised both natural and computer-generated voices that had been degraded, at rates significantly above chance. Kanzi performed better with all forms of natural voice speech compared to computer-generated speech. This work provides additional support for the hypothesis that the processing apparatus necessary to deal with highly variable speech, including for the first time in nonhuman animals, computer-generated speech, may be at least as old as the last common ancestor we share with bonobos and chimpanzees.

**Supplementary Information:**

The online version contains supplementary material available at 10.1007/s10071-022-01621-9.

## Introduction

The extent to which the human auditory processing system is specialized for speech is of major relevance for understanding the evolutionary origins of speech and language. Although a range of nonhumans can learn to understand nauralistic human speech, a substantial body of data from the field of speech science has indicated that humans are highly proficient at dealing with the potentially infinite multidimensional variability of the speech signal. One notable example comes from experimental work demonstrating that humans are capable of processing highly degraded speech, such as sine-wave speech or noise-vocoded speech, where essential cues to phonetic content are removed (cf. Fitch 2018). In sine-wave speech, the signal is reduced to the centre frequencies of the first three formants by replacing them with sinusoidal waves (frequency modulation) that track the formant structure in frequency and amplitude. With this, pitch cues to speech and most information about consonants is removed. On the other hand, noise-vocoded speech (the result of a procedure that is often used to simulate the hearing impression of listeners with cochlear implants), works by separating the speech signal into a set of distinct frequency bands, and using the amplitude envelope (ENV) of each band-limited signal to modulate white noise (see Hervais-Adelman et al. 2011). Whilst both forms of degraded speech sound unnatural or peculiar upon first hearing, they remain highly intelligible to both adults and children with relatively little training (Remez et al. 1981; Shannon et al. 1995; Friesen et al. 2001; Newman and Chatterjee 2013). One key implication of this speech-specific perceptual phenomenon is that it has been argued to provide support for the idea that the human auditory system is specialised for speech (Liberman 1957; Mattingly and Liberman 1988; Fitch 2011). The documented proficiency of some nonhumans at understanding naturalistic human speech (e.g. Savage-Rumbaugh et al. 1993; Kaminski et al. 2004) has been assumed to rely on general sound processing mechanisms that are qualitatively different from the specialised speech perception mechanisms humans are argued to possess (Heimbauer et al. 2011; Fitch 2018). Experimentally testing nonhuman abilities to process highly variable and degraded forms of speech is a key test of the argument for a species-specific speech processing mechanism in humans.

In a comparative study, Heimbauer et al. (2011) directly challenged this assumption of specialised speech processing in humans, presenting data suggesting a language competent chimpanzee was also capable of processing degraded human speech (in the forms of noise-vocoded and sine-wave speech). The authors argued these data provide support for the hypothesis that the mechanisms necessary for processing highly variable speech are evolutionarily more ancient than previously thought. Although this study provides important evidence to contest the assumption that the human auditory system is uniquely capable of processing variable and perturbed speech, further investigation is warranted. Firstly, data from other language competent great ape species can extend this work and provide a broader foundation for the phylogenetic reconstruction of the origins of speech processing mechanisms. Data from multiple closely-related ape species can serve to more convincingly rule out convergent or independent evolutionary origins as opposed to shared ancestry (Fitch 2017). Secondly, additional tests of the limits of great ape speech processing capabilities can be gleaned from exposure to novel, non-natural forms of speech, with one candidate being computer-generated speech. This speech is interesting because it reduces the signal to direct acoustic cues, and lacks cues containing information about the particular speaker and situational styles or more locally, co-articulatory information between sound segments that contain cues to the overall word identity.

Here we investigate the processing of degraded versions of both naturalistic human and computer-generated speech in a member of an equally closely related species to humans—Kanzi the language competent bonobo. Kanzi was raised in a language-enriched environment interacting with both humans and bonobos from an early age, and therefore has had extensive experience with human speech (Savage-Rumbaugh et al. 1993; Savage-Rumbaugh and Lewin 1994) and has demonstrated a rich understanding of the content of naturalistic human speech (Savage-Rumbaugh et al. 1993). Furthermore, he has been part of a larger research program aimed at investigating the capacity of our closest living relatives to understand and respond to human speech via an interactive system based on visuo-graphic symbols, otherwise known as lexigrams (Lyn et al. 2008). Over the last 4 decades, Kanzi has had continuous access to visual lexigram boards (some versions of which produced natural speech recordings of the item when pressed), and uses them daily to interact with caretakers (e.g. to request specific food items, or even games that he wishes to play by selecting lexigrams such as ‘water’ and ‘chase’). Moreover, at the time these data were collected, Kanzi’s understanding of the relative meaning of lexigram symbols in relation to human speech have been tested regularly using match-to-sample programs, which he completed approximately 1–3 times per week as part of his enrichment program (Rabinowitz 2016). We capitalised on Kanzi’s existing proficiency with touchscreens and lexigrams, and using a touchscreen-based match-to-sample paradigm, exposed him to both natural and computer-generated versions of 40 of his most familiar words that had been acoustically degraded (noise-vocoded and sine-wave forms). We also implemented an identical paradigm in a human sample to directly compare the abilities of a nonhuman subject to human subjects in their acoustic processing of speech samples. In line with Heimbauer et al. (2011), because sine-wave degradation processing in particular has been argued to stem from a specialised speech-related module in humans (Heimbauer et al. 2011; Trout et al. 2001), we predicted that Kanzi would have greater accuracy with noise-vocoded stimuli in comparison to sine-wave stimuli. We also predicted that Kanzi would perform better with degraded natural stimuli in comparison to degraded computer-generated stimuli sets, given that the latter stimuli are particularly distinct from natural speech, being both degraded and artificial, and since Kanzi has more experience with natural speech.

## Methods

### Subject and study site

Experiments were conducted at the Ape Cognition and Conservation Initiative, Iowa, USA. Kanzi was tested in his home enclosure while briefly (less than 60 min) separated from other group members.

### Procedure

All training and test words were taken from Kanzi’s existing vocabulary, and selected if he showed an accuracy of 80% or above during previous match-to-sample testing; resulting in 10 training samples and 40 test samples. Samples consisted of one-, two- and three-syllable words. In all trials, playback of the audio file was followed by the presentation of three lexigram choices. While this differed to the four lexigram choices Panzee was tested with (Heimbauer et al. 2011), this replicated previous match-to-sample testing formats that Kanzi had extensive and recent experience with (Rabinowitz 2016). Foil choices consisted of any words scoring 70% or above accuracy. These were randomly allocated to sample words in pairs, with each sample word having a unique foil pair. More information on the training procedure is provided in the Electronic Supplementary Material (ESM).

### Testing

Testing consisted of the following five conditions: *(1) Natural voice; (2) Sine-wave with a natural voice; (3) Noise-vocoded with a natural voice; (4) Sine-wave with a computer-generated voice; (5) Noise-vocoded with a computer-generated voice.* Kanzi was presented with each of the 40 test words, once in each of these conditions (*N* = 200 test samples, see below for stimulus generation). Test trials were randomly interspersed between filler trials (63–79% accuracy in previous match-to-sample; presented in a natural voice), with test trials occurring every 3–5 trials (see ESM). Due to an apparent effect of voice origin (natural or computer-generated), a further 3 testing sessions were created for an additional condition (**6**) *Computer-generated non-degraded * (*N* = 40). This consisted of unmanipulated audio stimuli of computer-generated speech. In total Kanzi completed a total of 19 test sessions (range 43–75 trials), with 18 sessions considered in analyses (see ESM).

### Stimulus generation

Noise-vocoded and sine-wave sound transformations used were developed by C. Darwin (www.lifesci.sussex.ac.uk/home/Chris_Darwin). Noise-vocoded words were created by first extracting seven frequency-limited energy bands from the original waveform, together spanning a range of 50 to 11,025 Hz (i.e., 50–800, 800–1500, 1500–2500, 2500–4000, 4000–6000, 6000–8500, and 8500–11,025). Amplitude envelopes were extracted from each of the resulting waveforms and used to temporally modulate corresponding band-limited white-noise. Sine-wave versions were made by frequency modulating three sinusoids with the corresponding frequency contour of the lowest three major resonances (formants) of the vocal tract. Each frequency modulated sinusoid was created individually and then summed for the final sine-wave speech. Formant contours were edited in places to produce the best possible match to formant tracks visible in spectrographic representations of each word. These sine waves were summed and amplitude normalized.

As computer-generated speech we chose formant synthesised speech in which both the source and the filter components as well as rudimentary prosodic information was fully generated by the use of digital-signal processing procedures. Unlike contemporary computer-generated speech in modern technical applications like smartphones which is most typically based on reprocessing human speech (concatenative synthesis, uni-selection or deep fake speech), formant synthesised speech lacks the naturalness of human-produced speech (speech sounds are maximally similar) and hence sounds most robotic-like. To create a computer-generated voice, the eSpeak NG formant synthesiser, implemented in the software Praat (www.praat.org) using a US English-speaking male. *Natural voice* conditions contained stimuli words spoken by a male human and which the participant was accustomed to hearing during his normal match-to-sample programs. Stimuli exemplars are available on Dryad (see Data Availability section).

### Human experiment

In contrast to previous work which implemented a human-specific control experiment (Heimbauer et al. 2011), we instead ran a highly similar perception experiment of human subjects’ recognition of manipulated and computer-generated speech (*N* = 11, see ESM). This included exposing our human subjects to the same training procedures as Kanzi to ensure that they were equally familiar with both degraded stimuli forms, and the format of the experiment prior to testing. Although arguably over-simple for participants, we reasoned this approach allowed a direct comparison with Kanzi’s performance. Given the nature of the task, we expected ceiling performance for human listeners for this task.

### Statistics

We analysed Kanzi’s performance on each condition using binomial tests. The maximum score Kanzi could achieve was 40/40 for each condition. Given Kanzi could choose between three options when making his selection we set the chance level at 0.33.

## Results

**Fig. 1:**
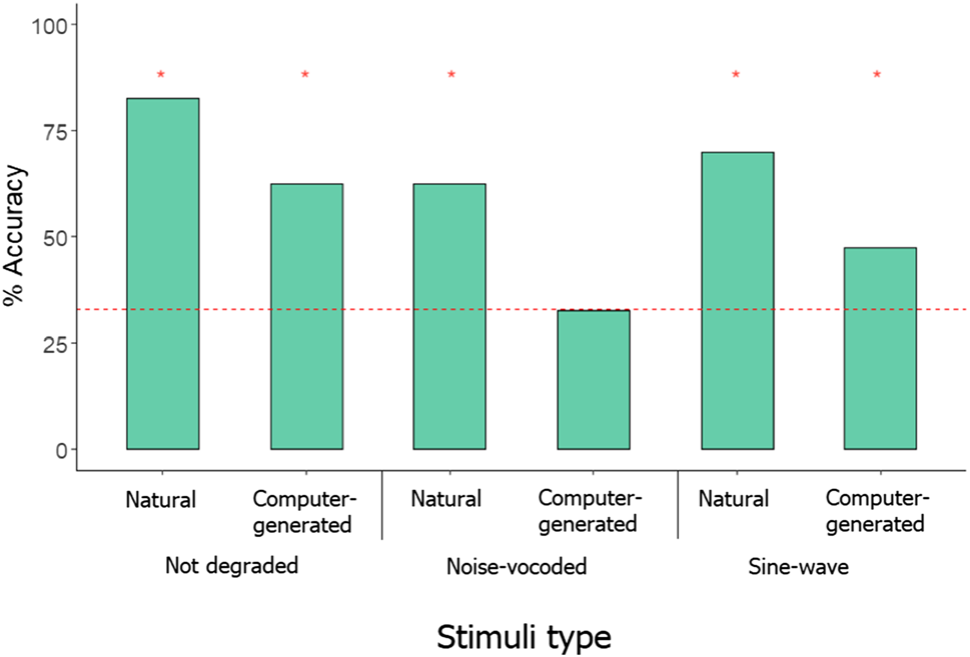
Kanzi’s performance accuracy across all stimulus types. Red line indicates chance level (33%) against which Kanzi’s performance in each condition was tested. *’s indicate that the corresponding binomial test was significantly above chance

Kanzi’s initial responses to degraded (sine-wave and noise-vocoded) versions of both natural and computer-generated stimuli that he encountered in the training sessions are reported in the ESM. In test trials, Kanzi was most accurate with unmanipulated, natural voice words, selecting the correct lexigram 83% of the time, a level significantly above chance (33/40, *p* < 0.001). Kanzi also chose the correct lexigram at a rate significantly higher than chance for both manipulated forms of natural voice stimuli (noise-vocoded: 25/40 trials correct, *p* < 0.001; sine-wave: 28/40 trials correct, *p* < 0.001). For the computer-generated stimuli, Kanzi’s performance decreased but remained significantly higher than chance for unmanipulated stimuli (25/40 trials correct, *p* < 0.001) and for sine-wave stimuli (19/40 trials correct, *p* = 0.040). For noise-vocoded computer-generated stimuli, however, Kanzi’s performance did not significantly differ from chance (13/40 trials correct, *p* = 0.55, see Fig. 1).

Additional analyses to explore the possibility that Kanzi was selecting responses based upon temporal cues (e.g. syllable number) rather than acoustic properties are also included in the ESM.

As expected, all human subjects interacting with the identical task reached ceiling or close-to ceiling levels for all conditions (mean % correct across all four degraded conditions: 99% and non-degraded computer-generated speech: 100%).

## Discussion

To our knowledge, prior to the current study, Kanzi had never been experimentally exposed to degraded forms of familiar words. Nonetheless, Kanzi was capable of correctly recognising them in a match-to-sample paradigm, picking the appropriate corresponding lexigram at a rate significantly higher than chance. Furthermore, we expanded upon Heimbauer et al.’s (2011) work by demonstrating for the first time that a nonhuman is capable of recognising not just degraded speech but also fully computer-generated speech. This type of speech lacks the naturalness and many alternative cues to word meaning, yet Kanzi continued to be able to recognise it even when it had undergone some forms of degradation. Although Kanzi’s performance with computer-generated speech was lower than with natural speech (both unmanipulated and degraded), he still chose the correct lexigram at a rate significantly higher than chance for the unmanipulated computer-generated stimuli and for the sinusoidally degraded versions of these stimuli. It is worth noting that the specific synthesis mode (formant synthesis) used for creating the computer-generated stimuli was overtly crude and un-human like (robotic-like) and previous work has shown that humans can struggle with such computer-generated stimuli, primarily when presented with novel phrases (Pisoni 1997). That Kanzi could still understand even some degraded versions of formant synthesised computer-generated stimuli demonstrates the existence of perceptual mechanisms in bonobos that are remarkably resilient when presented with highly deviant, non-natural speech.

Our findings support, but also subtly contrast with, previous work looking at the processing of degraded speech in a language competent chimpanzee, Panzee (Heimbauer et al. 2011). Similarly to Panzee, Kanzi performed best with natural, unmanipulated stimuli with a success rate comparable to his historical performance with the same words (91%). Like Panzee, Kanzi also scored significantly above chance on both noise-vocoded and sine-wave stimuli with a natural voice. Interestingly, however, we found differences regarding which form of degraded stimuli his performance was best with. Specifically, while Kanzi’s success rate was significantly higher than chance for both natural voice and computer-generated stimuli that had been sinusoidally manipulated, with noise-vocoded stimuli, Kanzi scored below chance for computer-generated voice stimuli variants. This finding is counter to what we initially predicted, and one potential explanation for this discrepancy might be attributable to the more tonal nature of the bonobo vocal communication system. In comparison to both chimpanzees and humans, bonobo vocalisations are generally up to an octave higher in pitch (Grawunder et al. 2018, though see also Garcia and Dunn 2019; Grawunder et al. 2019). It may therefore be that for bonobos, the underlying information content of sinusoidally manipulated words is inherently easier to abstract than for noise-based word degradation. However, given the results are derived from single individuals (in both chimpanzees and bonobos), follow up work replicating this effect in additional subjects is central to confirming this hypothesis.

We also ran a virtually identical experiment in humans to allow direct comparability between human and nonhuman subjects. As predicted, for all participants we saw ceiling effects, confirming that the tasks were trivial for human listeners. Generally, in human degraded-speech experiments, subjects are trained on degraded stimuli sets and then exposed to novel words or phrases which they are asked to accurately decipher (e.g. Heimbauer et al. 2011). In our experiment, subjects heard single words and in addition to this, were given 3 choices to make their selection from, which undoubtedly made the task considerably easier. Nevertheless, one advantage of the setup is that it nicely highlights, despite Kanzi’s success with parsing degraded speech, there still exists a gap that separates him from the humans we tested when dealing with such hyper-variable stimuli.

From a more general perspective, these results have interesting potential implications for our understanding of the evolutionary origins of speech. The fact that humans are capable of processing speech, even when it has been substantially perturbed (degraded) has, in the past, been argued to indicate that the perceptual mechanisms necessary for speech comprehension are unique to humans and distinct from general auditory processing (Remez et al. 1994, cf. Fitch 2011). Although comparative work also demonstrating degraded speech processing in chimpanzees is suggestive that these perceptual mechanisms may be rooted more deeply within the primate lineage (Heimbauer et al. 2011), additional data in other closely related ape species are important to making a stronger case for homology, and to more confidently rule out the possibility of convergence i.e. the same ability evolving independently in different species (Fitch 2017). Our work contributes to this debate by revealing very similar abilities in another species closely related to humans.

It should also be pointed out that, similarly to Panzee, Kanzi has had extensive experience with human speech over his lifetime, which undoubtedly played a role in his ability to parse degraded speech. However, whether experience alone is sufficient to explain the data remains unresolved. The next important step in understanding the relative contributions of phylogeny and experience to processing of highly variable speech is to run similar experiments with species more phylogenetically distantly related to humans, who also have experience with human speech. If the capacity for processing degraded speech stems more from experience and ontogenetic factors, we would expect species familiar with human speech (e.g. domesticated dogs) to share this ability with Panzee and Kanzi. An alternative approach to ascertain the influence of developmental and evolutionary processes would be to probe whether competence on such tasks varies as a function of age; if phylogeny plays a role in speech processing, then enculturated individuals, once initially trained to use lexigrams, should be able to succeed at recognising degraded forms. If experience also plays a role, we should then expect improved performance as a function of age.

In conclusion, we show that, similarly to humans and chimpanzees, bonobos are capable of processing both degraded and computer-generated speech. These data therefore provide critical further support for the theory that the perceptual mechanisms necessary for dealing with speech are not unique to humans, but rooted more deeply within the primate lineage, likely evolving before language or speech emerged.

## Supplementary Information

Below is the link to the electronic supplementary material.

Supplementary file1 (DOCX 20 KB)

## Data Availability

Stimuli exemplars and all raw data used for analysis are available to download from Dryad (URL: https://datadryad.org/stash/share/R759pD7TtnORnD8fVpjoGuOn98CSYkj73yLL233UNo).
