# Supplementary material for: Degraded and computer-generated speech processing in a bonobo
Source: Anim Cogn. 2022 May 20;25(6):1393–8. doi: 10.1007/s10071-022-01621-9 (PMC9652166; doi:10.1007/s10071-022-01621-9)
Supplement: Supplementary file 1 — Supplementary file1 (DOCX 20 KB) [file 10071_2022_1621_MOESM1_ESM.docx]

Electronic Supplementary material

*Methods*

*Training*

Given Kanzi was naïve to manipulated and synthetic voice stimuli, in line with previous work (Heimbauer et al. 2011), before testing we exposed Kanzi to training programs to help him learn that the test stimuli can be processed in the same way as unmanipulated human speech. All stimuli presented during training consisted of words spoken by a familiar speaker, which Kanzi was accustomed to hearing during his regular match-to-sample programs (‘natural’). A single session consisted of 10 non-test stimuli in their natural form presented twice, both in conjunction with 10 sine-wave manipulated and 10 noise-vocoded counterparts, resulting in 40 trials per session. Natural stimuli were always presented in paired trials, with the altered version either being presented in the trial directly after the natural sample (stage 1), or in the trial directly before the natural counterpart (stage 2). Whether Kanzi first completed natural sine-wave trials or natural noise-vocoded trials was counterbalanced across sessions. Kanzi completed two training sessions from each stage, totalling 4 sessions and 160 trials (40 noise-vocoded-, 40 sine-wave- and 80 natural samples, over 4 days). Kanzi received a food reward for every correct trial, and acoustic feedback in the form of high and low pitch sounds indicating correct or incorrect choices respectively.

Due to poor accuracy on sine-wave stimuli during stage two, an extra 20 trial session were completed (5 noise-vocoded-, 5 sine-wave- and 10 natural-samples). Criterion was met following this session (80%+). Following a 2-month gap in testing, a retraining period was necessary before testing was resumed. Retraining replicated the four initial training sessions, and also included the additional 20 trial session. For training, Kanzi therefore completed a total of 360 trials, with 90 sine-wave-, 90 noise-vocoded- and 180 natural-sample trials across both time periods.

*Testing*

We initially created five test sets for Kanzi to complete. Within a test set there were 40 test trials (one presentation of each test word) with 5/40 test trials assigned to each condition (conditions 1-5). The order of these test trials was then randomised, and 3-5 filler trials were inserted between each test word (N=153 filler trials per set). Each test set therefore consisted of a list of 193 trials. A single test session then comprised presenting Kanzi with a maximum of 75 trials from a test set, however some sessions comprised fewer trials due to the perceived motivation of the participant, with sessions being aborted in cases of distraction (range 43-75 trials). Irrespective of whether Kanzi successfully completed 75 trials or the session was aborted early, the next session always started in the test set list where the participant previously ended the session. For condition (6) *computer-generated non-degraded* we created an additional sixth test set (N= 40 test words, N=153 filler trials), but as this condition was added later this type of stimuli was consequently presented separately from the former stimuli types. Performance on test trials was never reinforced: no reinforcement sounds were played during test sessions, and as food rewards were still required to maintain motivation to participate, food rewards were given on the filler trial before the test trial (if correct), or the next correct filler trial after the test sample.

It took Kanzi a total of 18 test sessions to complete all 1,158 trials (240 trials with test items and 918 trials with filler items). One test session was repeated at the conclusion of testing, 4-weeks after it was initially presented to the participant, due to it initially being run prior to re-training in the second testing period. The second presentation of these trials was included for analysis.

*Initial response to stimuli*

As Kanzi heard each test word multiple times, it is potentially informative to understand how he responded to different types of degraded stimuli on first presentation. Upon very first exposure to sine-wave natural speech and noise-vocoded natural speech stimuli during training, Kanzi selected the correct lexigram for the first trial of both degraded speech types. Given that Kanzi was trained only on stimuli with the voice he was familiar with (‘natural’), his first exposure to degraded computer-generated stimuli occurred during test trials. Kanzi successfully identified the correct lexigram for his first trial with computer-generated non-degraded speech and computer-generated sine-wave synthetic speech, but made an incorrect selection for his first noise-vocoded computer-generated speech trial.

*Examination of potential temporal processing strategies*

It is possible that instead of using the acoustic properties of test words to correctly identify them Kanzi was cued by the syllable number or word duration of test words in comparison to foil choices. We therefore compared our 40 test words to the two foil choices they were paired with to examine this possibility. We reasoned that if Kanzi was relying on temporal cues, he should select the correct lexigram for the word in the majority of the six conditions, regardless of the type of degradation (as the degradation procedures did not affect the temporal structure of the words). For both syllable number and word duration strategies we examined (1) whether Kanzi selected the correct lexigram in the majority of presentations of each test word (minimum of 4/6 presentations), and (2) whether Kanzi consistently selected the correct lexigram for a test word (6/6).

To assess syllable number effects we compared cases where the test sample had a different number of syllables compared to either foil choice (N=22), with cases where the test sample had the same number of syllables as one or both foil choices (N=18). Kanzi selected the correct lexigram for a majority of word presentations for 13/22 words where the test word and foils had different numbers of syllables, compared to 6/18 words where the test word had the same number of syllables as at least one of the foils; a Fisher’s exact test revealed that these proportions were not significantly different (*p* = 0.13). Regarding the consistent selection of the correct lexigram option, a Fisher’s exact test revealed no significant difference in Kanzi’s performance when the test word and foils had different numbers of syllables (5/22), compared to when the test word had the same number of syllables as at least one of the foils (1/18; *p* = 0.20).

To assess word duration effects we then investigated whether his performance was lower on trials where the test word and foil word durations were more homogenous than heterogeneous (Heimbauer et al., 2011). We therefore calculated duration differences between the test sample and each of the two foil words and then calculated a mean of these two differences. We then ranked the mean duration differences for the forty test words. Mean duration differences falling below the median were then considered as having similar durations, and those falling above the median were considered as having more heterogenous word duration. When assessing whether Kanzi selected the correct lexigram in a majority of test word presentations, a Fisher’s exact test again showed no significant difference in Kanzi’s performance when test words and foils were similar in duration (7/20) than heterogenous in duration (12/20; *p* = 0.21). Finally, when assessing whether Kanzi selected the correct lexigram consistently for test word presentations, a Fisher’s exact test showed no significant difference in his performance when test words and foils were similar in duration (2/20) than heterogeneous in duration (4/20; *p* = 0.66).

*Human experiment*

Participants were all students at the University of York. As with Kanzi, participants were first exposed to a training period consisting of three repeated training stages (Stage 1=80 trials, stage 2 = 80 trials, stage 3= 40 trials, N=200 trials) of natural and manipulated (noise-vocoded and sine-wave) versions of Kanzi’s training stimuli, again to familiarise them with noise-vocoded and sine-wave speech. Subjects heard the stimuli and then were presented with the correct word and two foils on a computer monitor. In line with Kanzi, acoustic feedback in the form of high and low pitch sounds were used to indicate correct or incorrect choices respectively.

Within the same experimental session participants then moved immediately into the test period. Like Kanzi, subjects were exposed to the random presentation of Kanzi’s top 40 words in four degraded-stimuli conditions (**1**) s*ine-wave with a natural voice* (**2**) *noise-vocoded with a natural voice*; (3) *sine-wave with a computer-generated voice*; (**4**) *noise-vocoded with a computer-generated voice* (Test session 1: N trials=160)*.* However, given the predicted ease of participants recognising un-manipulated natural words, we did not expose subjects to all 40 natural words but instead randomly interspersed 20 of these as filler trials to maintain motivation. Finally, to verify that human subjects could spontaneously process computer-generated speech we exposed them to a fifth condition - a block of un-manipulated computer-generated versions of Kanzi’s top 40 words - at the end of the experiment (Test session 2: N trials = 40; total trials over test sessions 1 and 2 = 200).
